# Editorial: Explainable artificial intelligence for trustworthy and human-centric healthcare: methods, evaluation, and clinical impact

**DOI:** 10.3389/frai.2026.1990455

**Published:** 2026-09-23

**Authors:** Ionel Eduard Stan, Gregor Stiglic, Jose M. Juarez

**Affiliations:** 1 University of Milano-Bicocca, Milan, Italy; 2 University of Maribor, Maribor, Slovenia; 3 University of Murcia, Murcia, Spain

**Keywords:** explainable artificial intelligence, healthcare informatics, model transparency, counterfactual explanations, clinical decision support, fairness and bias, human_AI interaction

## From explanation to justified reliance

Artificial intelligence can support healthcare only when its outputs can be assessed in the context of the decisions they inform. A prediction may be accurate yet difficult to scrutinize; an explanation may be persuasive yet provide little evidence that the prediction is dependable. Explainable artificial intelligence (XAI) therefore raises a practical question: what must an explanation enable a clinician, patient, or healthcare organization to understand and do? Answering this question requires connecting computational methods with evaluation, clinical knowledge, and the circumstances of use.

This Research Topic brings together 15 contributions addressing that connection across clinical prediction, imaging, physiological signals, biomedical networks, language models, rehabilitation, and healthcare processes. Collectively, the papers illustrate an expanding range of approaches to transparency. They also suggest that trustworthy and human-centric healthcare requires more than the availability of an explanation: it requires evidence that explanations support appropriate reliance, including the ability to question an output and recognize its limitations.

Two systematic reviews establish this perspective. Aravindkumar et al. synthesize 36 studies across imaging, diagnosis, and rehabilitation, identifying recurring relationships between explanation methods and clinical tasks alongside gaps in validation and reporting. Din et al., reviewing 51 studies involving healthcare professionals in medical imaging, distinguish favorable perceptions of explanations from improvements in diagnostic performance. Their findings caution against treating perceived clarity or increased confidence as sufficient evidence of clinical usefulness. The review also identifies promising signals for example-based and concept-based explanations, while emphasizing the limitations of comparisons across heterogeneous studies.

## Making predictions and representations inspectable

Several contributions apply feature attribution to clinically relevant prediction problems. Chen et al. use an 11-year, single-center cohort of 1,062 patients to predict postoperative ileus after radical cystectomy, combining machine learning with global and patient-level Shapley additive explanations (SHAP). The study illustrates how routinely collected perioperative information can support interpretable risk summaries, while its retrospective design and absence of external validation leave portability and clinical benefit to be established.

Paul and Syed Masood examine cardiovascular prediction using structured health data, comparing models and explaining feature contributions with SHAP. Ghosh et al. investigate reproductive tract infection risk using survey information from a Delhi college-based cohort. Together, these studies demonstrate the accessibility of attribution methods for structured data. Their findings also underline the importance of defining the prediction target and population: cross-sectional classification and self-reported symptoms have different evidentiary implications from longitudinal risk prediction and laboratory-confirmed disease. Feature importance should be interpreted within those boundaries.

Other papers connect explanations with domain-specific representations. Zhao et al. combine tongue and facial image features for coronary artery disease classification, using feature gating and SHAP. Their external test set adds evidence beyond internal validation, although the case–control design and population characteristics warrant prospective evaluation in more diverse settings. Sun et al. introduce scale-consistent attribution for heart sound classification, incorporating physiological frequency priors during training. This approach makes domain knowledge part of model development; whether stronger prior alignment improves clinical usability remains an open empirical question.

Wang and Qin integrate feature extraction, a corneal knowledge graph, probabilistic inference, and language-based reporting in a neuro-symbolic framework. Their 20-eye pilot includes specialist assessment of interpretability, offering an initial connection between technical design and user evaluation. At a different biological scale, Maruhashi et al. introduce truncated reconstruction based interpretable prediction (TRIP) to organize families of causal graphs into interpretable latent mechanisms, with synthetic experiments and cancer cell-line network analysis. This contribution extends explainability toward scientific hypothesis generation, whose validity remains linked to the assumptions and quality of the underlying graphs.

## Grounding generated explanations in evidence

Language-based systems create opportunities to communicate complex information, but readable output requires a verifiable evidentiary basis. Alu and Oluwadare address this requirement through a conceptual architecture combining retrieval-augmented generation, source provenance, and tamper-evident audit logging. The contribution is a design framework, with implementation and evaluation still needed. Its emphasis on recording the evidence behind recommendations gives transparency an organizational dimension: users should be able to inspect where information originated and how it entered the decision process.

Dindukurthi et al. provide a complementary implementation for personalized nutrition recommendations. Their graph-based framework represents disease–nutrient–food relationships and demographic requirements, while restricting language generation to formatting graph-validated outputs. Controlled evaluations support the proposed architecture, but expert and clinical validation remain future work. The design illustrates a useful division of responsibilities between structured knowledge, explicit computation, and natural-language presentation.

Zhang et al. evaluate reasoning-capable language models using qualitative and quantitatively encoded thyroid ultrasound descriptors. Differences across models and frequent inconsistencies between the two input representations highlight the need to test reproducibility and consistency alongside accuracy. Because the inputs are structured text rather than raw ultrasound images, the results concern reasoning over described features. Clinician-rated reasoning quality should also be distinguished from evidence that generated rationales faithfully reveal a model's internal computation.

## Evaluating people, processes, and fairness

The Research Topic also broadens the settings in which human-centric evaluation matters. Bellini et al. introduce TEXLoS, combining patient-pathway classification, feature attribution, and process discovery to investigate associations between services delivered during hospitalization and length of stay. An initial evaluation with six end users connects the explanations to organizational understanding. These associations can guide investigation of bottlenecks, although they do not establish that changing a service will shorten hospitalization.

Boonyaratana et al. assess a real-time AI neck exercise program in 30 healthy young adults. Their findings of moderate measurement reliability emphasize a complementary requirement for trustworthy systems: feedback must rest on dependable measurement. This study contributes evidence about validity and reliability rather than demonstrating that explanations improve rehabilitation outcomes. It also illustrates why evaluation should reflect the intended population and operating environment.

Fairness requires a similarly distinct assessment. Alu et al. use a synthetic patient dataset to compare models within a provenance-based auditing framework. The experiment shows why model interpretability cannot be assumed to guarantee equitable behavior. Its proposed fairness record links data origins, modeling choices, and disparity measures, making auditing more explicit. The simulation offers a methodological demonstration; real-world fairness and the adequacy of any governance process require evaluation in the relevant clinical setting.

## An agenda for clinical impact

Taken together, these contributions suggest three priorities. First, explanation claims should match the evidence. Predictive discrimination, physiological plausibility, explanation faithfulness, user comprehension, and patient benefit are different properties. Demonstrating one should motivate, rather than substitute for, evaluation of the others. In particular, an attribution identifies a contribution to a model output; it does not by itself identify a causal mechanism or an effective intervention.

Second, evaluations should specify the user, task, and decision that the explanation is intended to support. Where feasible, studies should compare human performance with and without explanations while holding the underlying AI predictions constant. Outcomes should include error detection, appropriate acceptance and rejection of advice, time requirements, and workload, alongside subjective assessments. Patient participation is also needed when explanations are intended to inform shared decisions or self-management.

Third, clinical translation requires external and prospective validation, attention to subgroup performance, and documentation of data and knowledge provenance. For generative systems, source support and consistency across inputs deserve explicit testing. For models incorporating expert priors, evaluation should examine circumstances in which those priors are incomplete or inappropriate. These priorities follow from the Research Topic's varied contributions and evidence gaps; they are an agenda for further investigation rather than outcomes already demonstrated across the Research Topic.

This Research Topic advances the discussion from how explanations can be produced toward how their value can be established. Its methodological diversity is a strength, provided that claims remain proportionate to the evidence. The next step is to connect inspectable models and traceable information with demonstrably better human decisions. In healthcare, the most valuable explanation will be one that helps people exercise informed judgment, including knowing when further evidence is needed.

